# Crystal structure and Hirshfeld surface analysis of anhydrous salt of levofloxacin and 4-methyl­benzoic acid

**DOI:** 10.1107/S2056989025010047

**Published:** 2025-12-14

**Authors:** Bhumi C. Patel, Krunal M. Modi, J. Prakasha Reddy

**Affiliations:** a Department of Chemistry, School of Sciences, Indrashil University, Rajpur, 382740, India; b School of Applied Material Sciences Central University of Gujarat, Vadodara 391107 India; Universidade Federal do ABC, Brazil

**Keywords:** crystal structure, levofloxacinium 4-methyl­benzoate salt, Hirshfeld, X-ray diffraction, hydrogen-bonding inter­actions

## Abstract

The anhydrous salt levofloxacinium 4-methyl­benzoate, C_18_H_21_FN_3_O_4_^+^·C_8_H_7_O_2_^−^, has been synthesized and its crystal structure determined. In the crystal, the levofloxacinium ions inter­act with the 4-methyl­benzoate anion *via* N—H^+^⋯O^−^ and C—H⋯O hydrogen bonds, forming a tape-like supra­molecular structure. Hirshfeld surface analysis and the calculated two-dimensional finger plots of the various atom–atom contacts involving both the ions are described.

## Abstract 2

In the crystal of the anhydrous salt of levofloxacin with 4-methyl­benzoic acid, C_18_H_21_FN_3_O_4_^+^·C_8_H_7_O_2_^−^, the levofloxacinium ions inter­act with the 4-methyl­benzoate anion *via* N—H^+^⋯O^−^ and C—H⋯O hydrogen bonds, forming a tape-like supra­molecular structure.

## 1. Chemical context

Small organic mol­ecules and peptides have been known for decades for their aesthetic supra­molecular architectures (Prabhakaran *et al.*, 2009 ▸; Upadhye *et al.*, 2009 ▸) and various applications including in the pharmaceutical industry (Shah *et al.*, 2023 ▸; Karmakar *et al.*, 2025 ▸; Gellman, 1998 ▸). Fluoro­quinolones belonging to a class of broad-spectrum anti­biotics having advantageous pharmacokinetic properties and are used in the treatment of various bacterial infections. Levofloxacin, (*levo* isomer of oflaxacin), systematic name: *S*-(−)-9-fluoro-2,3-di­hydro-3-methyl-10-(4-methyl-1-piperazin­yl)-7-oxo-7*H*-pyri­dine­[1,2,3-*de*]-1,4-benzoxazine-6-carb­oxy­lic acid, C_18_H_20_FN_3_O_4_, is a fluorinated third-generation fluoro­quinolone anti­biotic employed in the treatment of respiratory, urinary tract, cutaneous allergy and various other infections caused by Gram-positive and Gram-negative bacteria. A therapeutic review discussing the pharmacology, pharmacokinetics, *in vitro* activity, drug inter­actions, and adverse effects of levofloxacin has been published (Wimer *et al.*, 1998 ▸) and the use of levofloxacin in the treatment of community-acquired pneumonia was described (Noreddin *et al.*, 2010 ▸). A cohort analysis describing levofloxacin dosage to treat bone and joint infections was reported (Asseray *et al.*, 2016 ▸). A literature review of the levofloxacin in veterinary medicine was published recently wherein levofloxacin MIC values of animal microbial isolates are summarized (Sitovs *et al.*, 2021 ▸). A review on data summarizing the efficacy and the tolerability of levofloxacin in treating complicated urinary tract infections (UTIs) and pyelonephritis was described (Bientinesi *et al.*, 2020 ▸), as well as a review of levofloxacin for the treatment of bacterial infections (Noel, 2009 ▸) has also been published. Recently, a retrospective observational study of the efficacy and safety of levofloxacin in children with severe infections was conducted (Junqi *et al.*, 2024 ▸). Recently, levofloxacinium citrate salt hydrate (Nugrahani *et al.*, 2024 ▸) was reported, the crystal structure of which features O—H⋯O, N—H⋯O and C—H⋯O inter­actions. Various solvates of levofloxacin and its citrate salt have also been reported (Nugrahani *et al.*, 2022 ▸) wherein improvement in the anti­biotic potency and an anti­biotic–anti­oxidant combination for drug dosage development was reported. A study involving salts of levofloxacin with 2,6- and 3,5-di­hydroxy­benzoic acid showed increased stability and anti­biotic potency improvement (Nugrahani *et al.*, 2023 ▸). More recently, a drug–drug salt of levofloxacin flufenamic acid was reported along with its physicochemical properties, potency and anti-inflammation improvements that could be developed further into dosage formulations (Nugrahani *et al.*, 2025 ▸).

The preparation of anhydrous forms of levofloxacin, salts or co-crystals (Freitas *et al.*, 2018 ▸; Wei *et al.*, 2019 ▸) continues to be challenging as these anhydrous forms readily convert into hemihydrate/hydrate forms (Singh *et al.*, 2014 ▸). Continuing our research in the area of co-crystals (*e.g.* PrakashaReddy *et al.*, 2004 ▸), we herein report the synthesis of a new anhydrous levofloxacinium:4-methyl­benzoate salt, (I). We have determined its mol­ecular and crystal structures and conducted a Hirshfeld surface analysis to examine the inter­molecular inter­actions.
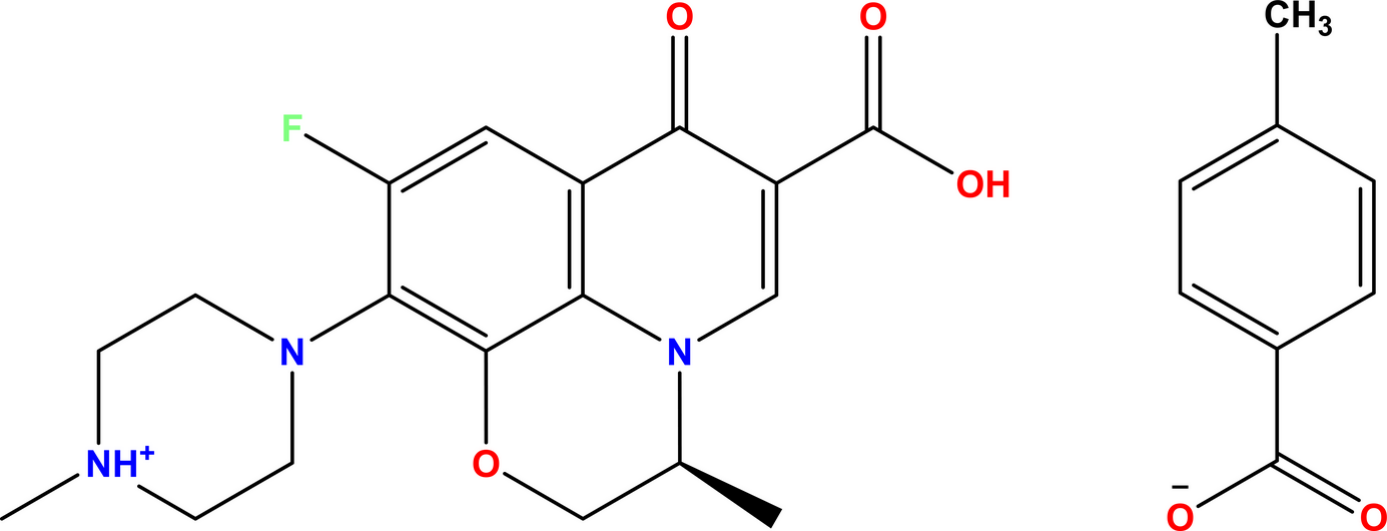


## 2. Structural commentary

**Figure 1:**
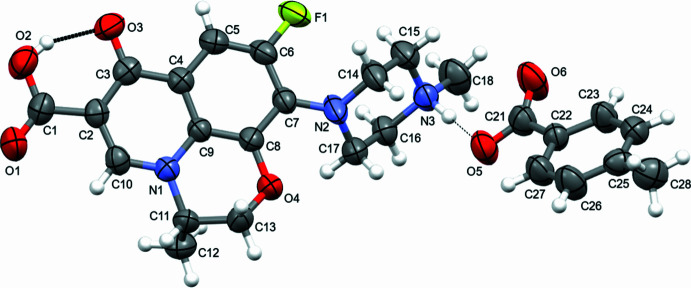
The mol­ecular structure of the levofloxacinium:4-methyl­benzoate salt, showing the atom labelling and displacement ellipsoids drawn at the 30% probability level. Intra­molecular hydrogen bonds are drawn as thick dashed lines while inter­molecular hydrogen bonds are drawn as thin dashed lines.

Reaction between levofloxacin and 4-methyl­benzoic acid yielded the title salt, (I), which crystallizes in the ortho­rhom­bic *P*2_1_2_1_2_1_ space group with one ion pair in the asymmetric unit. The mol­ecular structure of the salt along with the atom-labelling is shown in Fig. 1 ▸. The quinoline ring along with the other attached carboxyl and fluorine atoms in the levofloxacinium are essentially planar (r.m.s. deviation = 0.0659 Å), as observed in another salt hydrate reported in the literature (Golovnev *et al.*, 2018 ▸). On the other hand, the carboxyl­ate group in the 4-methyl­benzoate is twisted notably from the planarity of methyl aromatic plane with a torsion angle of −18.1 (8)^o^ for the chain of O5—C21—C22—C27 atoms. An intra­molecular O2—H2⋯O3 hydrogen bond is observed between the hy­droxy O atom of the –COOH group and the adjacent quinoline oxygen atom, forming an *S*(6) ring motif, as seen in other salts/co-crystals of levofloxacin reported in the literature (Nugrahani *et al.*, 2022 ▸).

## 3. Supra­molecular features

**Figure 2:**
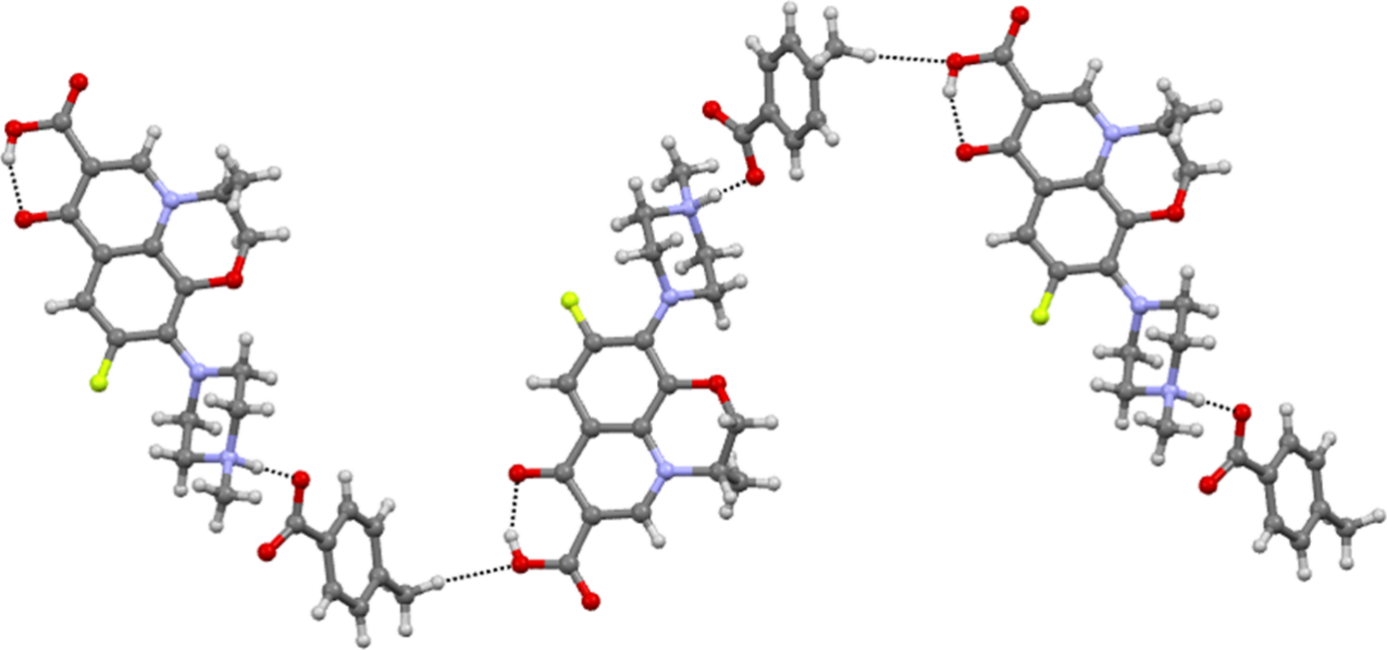
Formation of supra­molecular tape-like structure through N—H^+^⋯O^−^ and C—H⋯O inter­actions in the crystal.

**Figure 3:**
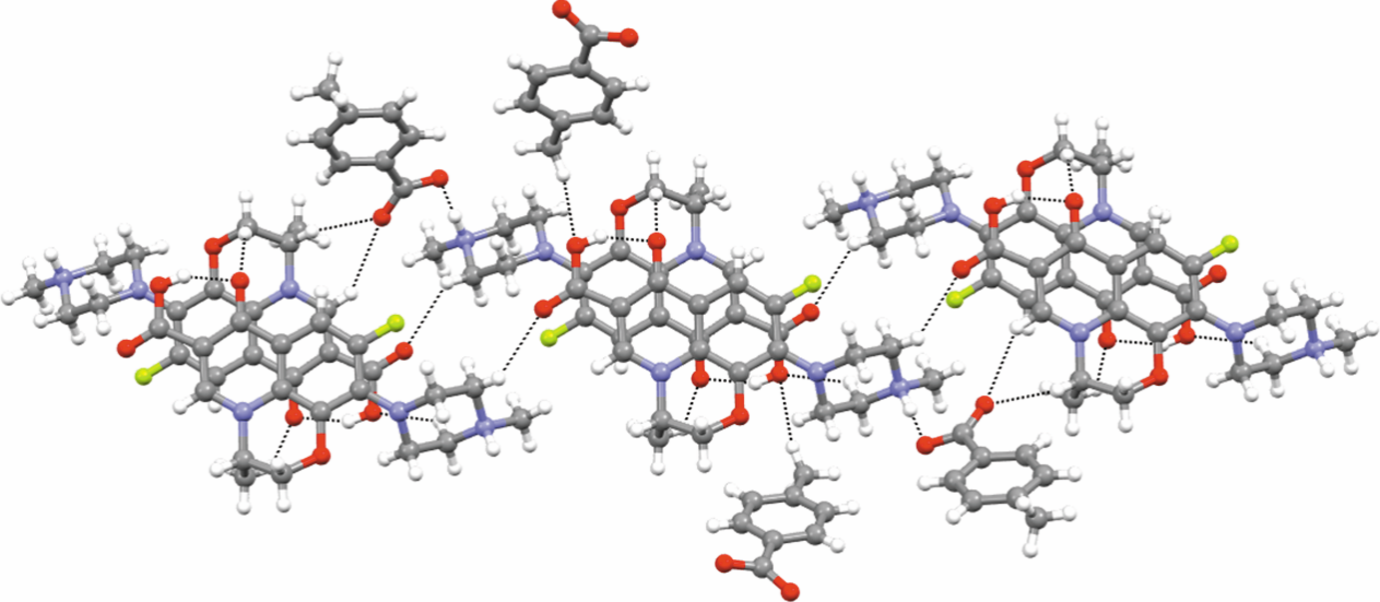
Various other C—H⋯O inter­actions observed in the crystal.

**Figure 4:**
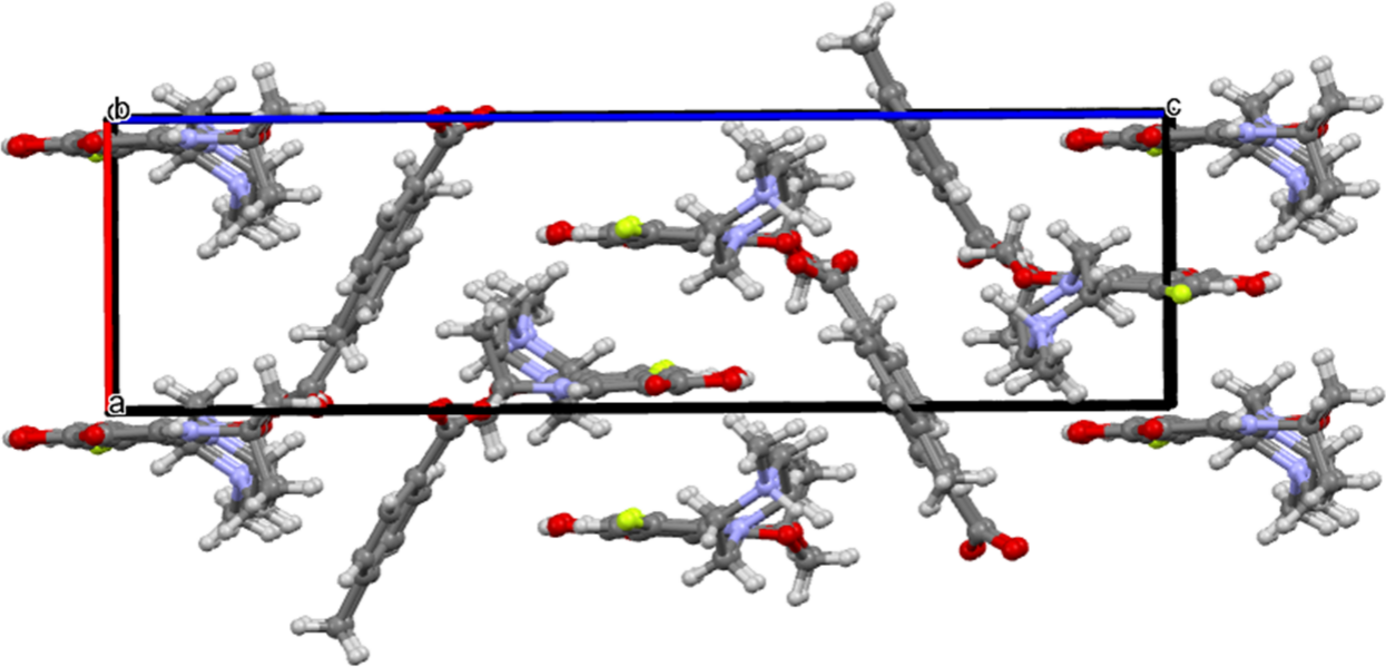
Three-dimensional packing viewed along the b-axis direction.

In the crystal, inter­molecular hydrogen-bonding inter­actions are observed. Levofloxacinium and 4-methyl­benzoate ions are connected through the N5—H6⋯O8 inter­action (Table 1 ▸). Further, a hydrogen atom of the methyl group of 4-methyl­benzoate inter­acts with the hy­droxy group –COOH of the levofloxacinium cation through the C28—H28*B*⋯O2 hydrogen bond (Desiraju, *et al.*, 1999 ▸; Patel, *et al.*, 2024 ▸), forming a tape-like supra­molecular structure as shown in Fig. 2 ▸. In addition, a number of other C—H⋯O inter­actions (C16—H16*B*⋯O2, C12—H12*C*⋯O3, C11—H11⋯O6, C15—H15*B*⋯O1, C10—H10⋯O6, C13—H13*A*⋯O3) between levofloxacinium ions and both levofloxacinium and 4-methyl­benzoate are observed in the crystal structure as shown in Fig. 3 ▸. The three-dimensional projection along the crystallographic *b*-axis is shown in Fig. 4 ▸.

## 4. Hirshfeld surfaces and 2D fingerprint plots

**Figure 5:**
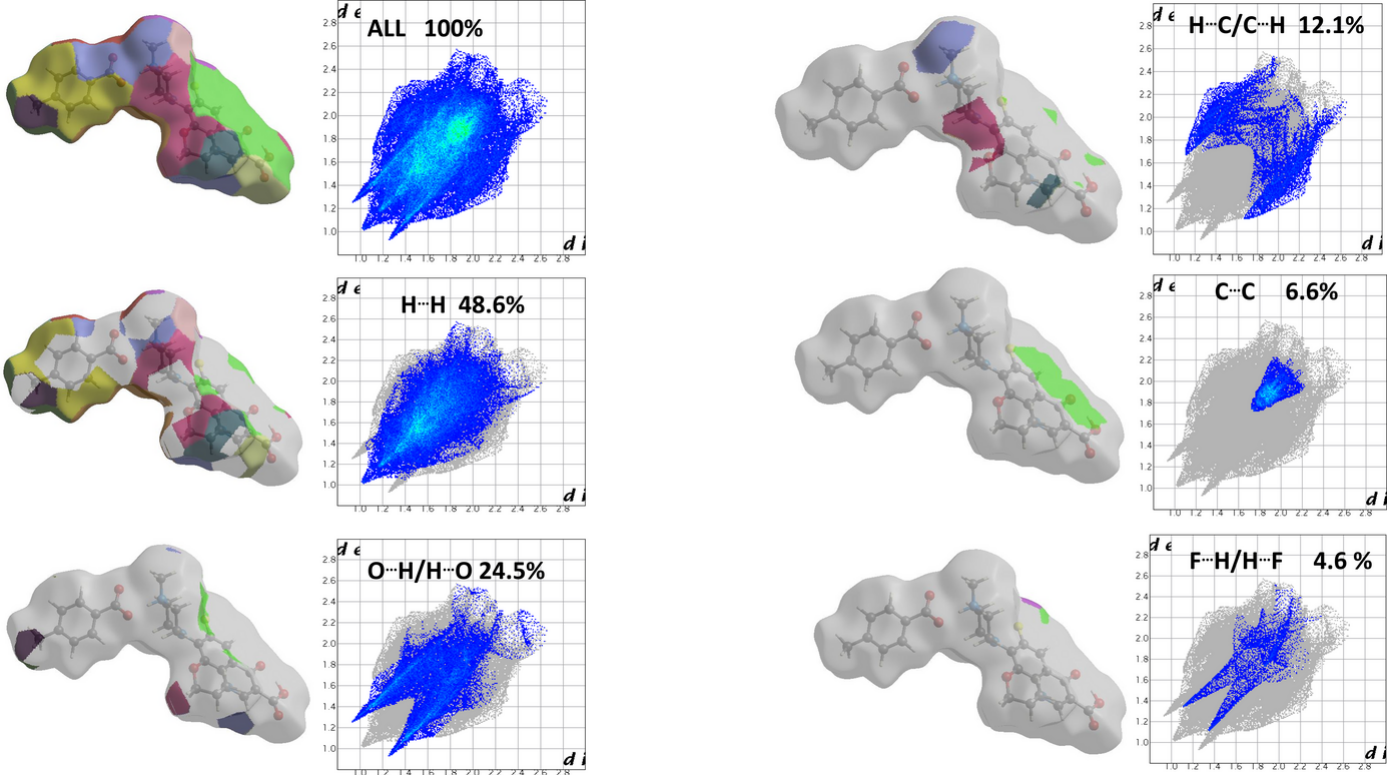
Hirshfeld surfaces of title compound mapped with *d*_norm_ (left image of each pair) and the corresponding two-dimensional fingerprint plots (right image of each pair) showing all contributions and then the major contributions of H⋯H followed by O⋯H/H⋯O, C⋯H/H⋯C, C⋯C and F⋯H/H⋯F contacts.

A Hirshfeld surface analysis and corresponding fingerprint plots were generated using *CrystalExplorer* software (Spackman *et al.*, 2021 ▸; Spackman & Jayatilaka, 2009 ▸) to further investigate and determine the contributions of the several inter­molecular inter­actions in the crystal. The Hirshfeld surface mapped over *d*_norm_ with the corresponding two-dimensional fingerprint plots (McKinnon *et al.*, 2007 ▸) for all inter­molecular inter­actions and those delineated into specific contacts are shown in Fig. 5 ▸. The largest contribution comes from H⋯H contacts at 48.6% of the total, which is consistent with the significant hydrogen content of the mol­ecule. The next most important contact is O⋯H/H⋯O at 24.5%, which primarily comes from the intra­molecular O—H⋯O and inter­molecular N—H⋯O as well as C—H⋯O inter­actions. The C⋯H/H⋯C inter­actions account for 12.1% while C⋯C contacts contribute 6.6%, followed by F⋯H/H⋯F contacts contributing 4.6%.

## 5. Synthesis and crystallization

Levofloxacin and 4-methyl­benzoic acid were obtained from Aldrich, and HPLC grade methanol was used for reaction. Levofloxacin (100 mg, 0.277 mmol) was dissolved in methanol (10 ml) under constant stirring at 335 K for 40 min. Equimolar solution of 4-methyl­benzoic acid (38 mg, 0.277 mmol) in methanol (10 ml) was added to the solution of levofloxacin and stirring was continued further for about 30 min at 335 K. The mixture was cooled to room temperature and the solution was filtered. X-ray quality single crystals of suitable dimension were obtained over a period of ten days by slow evaporation of the solvent.

## 6. Refinement

Crystal data, data collection and structure refinement details are summarized in Table 2 ▸. All hydrogen atoms were placed at idealized positions and refined using a riding model. The assignment of the absolute configuration is based on levofloxacin.

## Supplementary Material

Crystal structure: contains datablock(s) I. DOI: 10.1107/S2056989025010047/ee2021sup1.cif

Structure factors: contains datablock(s) I. DOI: 10.1107/S2056989025010047/ee2021Isup2.hkl

Supporting information file. DOI: 10.1107/S2056989025010047/ee2021Isup3.cml

CCDC reference: 2502089

Additional supporting information:  crystallographic information; 3D view; checkCIF report

## Figures and Tables

**Table 1:** Hydrogen-bond geometry (Å, °)

*D*—H⋯*A*	*D*—H	H⋯*A*	*D*⋯*A*	*D*—H⋯*A*
O2—H2⋯O3	0.82	1.76	2.522 (6)	155
N3—H3⋯O5	0.98	1.63	2.610 (6)	175
N3—H3⋯O6	0.98	2.51	3.125 (6)	121
C11—H11⋯O6^i^	0.98	2.28	3.147 (6)	147
C12—H12*C*⋯O3^ii^	0.96	2.52	3.308 (8)	139
C13—H13*A*⋯O3^iii^	0.97	2.40	3.302 (8)	154
C14—H14*B*⋯F1	0.97	2.30	2.785 (6)	110
C15—H15*B*⋯O1^iv^	0.97	2.58	3.268 (7)	128
C16—H16*B*⋯O2^ii^	0.97	2.55	3.277 (7)	131
C18—H18*C*⋯F1^v^	0.96	2.54	3.279 (8)	134
C28—H28*B*⋯O2^vi^	0.96	2.60	3.481 (7)	153

Symmetry codes: (i) 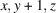; (ii) 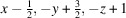; (iii) 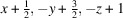; (iv) 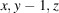; (v) 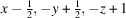; (vi) 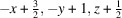.

**Table 2:** Experimental details

Crystal data	
Chemical formula	C_18_H_21_FN_3_O_4_^+^·C_8_H_7_O_2_^−^
*M*_r_	497.51
Crystal system, space group	Orthorhombic, *P*2_1_2_1_2_1_
Temperature (K)	120
*a*, *b*, *c* (Å)	7.1788 (10), 13.0274 (13), 25.979 (3)
*V* (Å^3^)	2429.6 (5)
*Z*	4
Radiation type	Mo *K*α
μ (mm^−1^)	0.10
Crystal size (mm)	0.39 × 0.29 × 0.23

Data collection	
Diffractometer	Bruker SMART APEXII CCD
Absorption correction	Analytical (*SADABS*; Krause *et al.*, 2015 ▸)
*T*_min_, *T*_max_	0.575, 0.746
No. of measured, independent and observed [*I* > 2σ(*I*)] reflections	17036, 5409, 2456
*R*_int_	0.116
(sin θ/λ)_max_ (Å^−1^)	0.644

Refinement	
*R*[*F*^2^ > 2σ(*F*^2^)], *wR*(*F*^2^), *S*	0.060, 0.177, 1.00
No. of reflections	5409
No. of parameters	330
H-atom treatment	H-atom parameters constrained
Δρ_max_, Δρ_min_ (e Å^−3^)	0.23, −0.17
Absolute structure	Flack *x* determined using 707 quotients [(*I*^+^)−(*I*^−^)]/[(*I*^+^)+(*I*^−^)] (Parsons *et al.*, 2013 ▸)
Absolute structure parameter	0.02 (10)

Computer programs: *APEX2* (Bruker, 2005 ▸), *SAINT* (Bruker, 2017 ▸), *SHELXT2018/2* (Sheldrick, 2015*a* ▸), *SHELXL2019/3* (Sheldrick, 2015*b* ▸) and *OLEX2* (Dolomanov *et al.*, 2009 ▸).

## supplementary crystallographic information

### 4-{11-Carboxy-7-fluoro-2-methyl-10-oxo-4-oxa-1-azatricyclo[7.3.1.0{5,13}]trideca-5,7,9(13),11-tetraen-6-yl}-1-methylpiperazin-1-ium 4-methylbenzoate Crystal data

**Table:**

C_18_H_21_FN_3_O_4_^+^·C_8_H_7_O_2_^−^	*D*_x_ = 1.360 Mg m^−^^3^
*M**_r_* = 497.51	Mo *K*α radiation, λ = 0.71073 Å
Orthorhombic, *P*2_1_2_1_2_1_	Cell parameters from 3952 reflections
*a* = 7.1788 (10) Å	θ = 2.5–27.5°
*b* = 13.0274 (13) Å	µ = 0.10 mm^−^^1^
*c* = 25.979 (3) Å	*T* = 120 K
*V* = 2429.6 (5) Å^3^	Irregular, clear whiteish colourless
*Z* = 4	0.39 × 0.29 × 0.23 mm
*F*(000) = 1048	

### 4-{11-Carboxy-7-fluoro-2-methyl-10-oxo-4-oxa-1-azatricyclo[7.3.1.0{5,13}]trideca-5,7,9(13),11-tetraen-6-yl}-1-methylpiperazin-1-ium 4-methylbenzoate Data collection

**Table:**

Bruker SMART APEXII CCD diffractometer	2456 reflections with *I* > 2σ(*I*)
Radiation source: fine-focus sealed xray tube	*R*_int_ = 0.116
ω scans	θ_max_ = 27.2°, θ_min_ = 1.8°
Absorption correction: analytical (SADABS; Krause *et al.*, 2015)	*h* = −9→9
*T*_min_ = 0.575, *T*_max_ = 0.746	*k* = −16→14
17036 measured reflections	*l* = −33→33
5409 independent reflections	

### 4-{11-Carboxy-7-fluoro-2-methyl-10-oxo-4-oxa-1-azatricyclo[7.3.1.0{5,13}]trideca-5,7,9(13),11-tetraen-6-yl}-1-methylpiperazin-1-ium 4-methylbenzoate Refinement

**Table:**

Refinement on *F*^2^	Hydrogen site location: inferred from neighbouring sites
Least-squares matrix: full	H-atom parameters constrained
*R*[*F*^2^ > 2σ(*F*^2^)] = 0.060	*w* = 1/[σ^2^(*F*_o_^2^) + (0.0526*P*)^2^] where *P* = (*F*_o_^2^ + 2*F*_c_^2^)/3
*wR*(*F*^2^) = 0.177	(Δ/σ)_max_ = 0.001
*S* = 1.00	Δρ_max_ = 0.23 e Å^−^^3^
5409 reflections	Δρ_min_ = −0.17 e Å^−^^3^
330 parameters	Absolute structure: Flack *x* determined using 707 quotients [(*I*^+^)-(*I*^-^)]/[(*I*^+^)+(*I*^-^)] (Parsons *et al.*, 2013)
0 restraints	Absolute structure parameter: 0.02 (10)
Primary atom site location: dual	

### 4-{11-Carboxy-7-fluoro-2-methyl-10-oxo-4-oxa-1-azatricyclo[7.3.1.0{5,13}]trideca-5,7,9(13),11-tetraen-6-yl}-1-methylpiperazin-1-ium 4-methylbenzoate Special details

**Table:**

Geometry. All esds (except the esd in the dihedral angle between two l.s. planes) are estimated using the full covariance matrix. The cell esds are taken into account individually in the estimation of esds in distances, angles and torsion angles; correlations between esds in cell parameters are only used when they are defined by crystal symmetry. An approximate (isotropic) treatment of cell esds is used for estimating esds involving l.s. planes.

### 4-{11-Carboxy-7-fluoro-2-methyl-10-oxo-4-oxa-1-azatricyclo[7.3.1.0{5,13}]trideca-5,7,9(13),11-tetraen-6-yl}-1-methylpiperazin-1-ium 4-methylbenzoate Fractional atomic coordinates and isotropic or equivalent isotropic displacement parameters (Å^2^)

**Table:**

	*x*	*y*	*z*	*U*_iso_*/*U*_eq_	
F1	0.3846 (6)	0.4254 (2)	0.48601 (11)	0.0839 (13)	
O4	0.4409 (7)	0.6279 (2)	0.63599 (12)	0.0654 (13)	
O3	0.4055 (6)	0.7860 (3)	0.41864 (13)	0.0690 (12)	
N1	0.4331 (7)	0.8049 (3)	0.57537 (14)	0.0507 (12)	
O5	0.5049 (7)	0.1973 (2)	0.69163 (16)	0.0809 (14)	
O2	0.4106 (8)	0.9794 (3)	0.41499 (15)	0.0817 (14)	
H2	0.412608	0.918689	0.406726	0.123*	
O6	0.5051 (8)	0.0488 (3)	0.65068 (18)	0.1007 (18)	
N3	0.2677 (7)	0.2393 (3)	0.61849 (16)	0.0592 (14)	
H3	0.351504	0.221625	0.646927	0.071*	
N2	0.4162 (8)	0.4350 (3)	0.59214 (17)	0.0709 (16)	
O1	0.4224 (8)	1.0696 (3)	0.48648 (15)	0.0909 (17)	
C3	0.4150 (8)	0.7922 (4)	0.46730 (19)	0.0529 (14)	
C2	0.4216 (8)	0.8873 (3)	0.49351 (19)	0.0510 (14)	
C4	0.4170 (8)	0.7005 (3)	0.49911 (19)	0.0482 (13)	
C7	0.4161 (9)	0.5228 (3)	0.5618 (2)	0.0530 (15)	
C9	0.4225 (8)	0.7085 (3)	0.55283 (17)	0.0469 (13)	
C8	0.4276 (8)	0.6204 (3)	0.58383 (17)	0.0505 (14)	
C10	0.4327 (8)	0.8895 (4)	0.54619 (19)	0.0546 (15)	
H10	0.440276	0.952924	0.562442	0.066*	
C11	0.4308 (10)	0.8137 (3)	0.63186 (17)	0.0558 (16)	
H11	0.502388	0.874645	0.641728	0.067*	
C6	0.4047 (9)	0.5197 (4)	0.5082 (2)	0.0577 (16)	
C16	0.1705 (9)	0.3364 (3)	0.6321 (2)	0.0596 (16)	
H16A	0.101626	0.326800	0.663871	0.072*	
H16B	0.082101	0.353582	0.605224	0.072*	
C22	0.7367 (9)	0.0768 (4)	0.71312 (19)	0.0565 (16)	
C5	0.4083 (9)	0.6029 (4)	0.4772 (2)	0.0574 (16)	
H5	0.405122	0.595200	0.441643	0.069*	
C1	0.4181 (10)	0.9871 (4)	0.4657 (2)	0.0651 (18)	
C25	1.0649 (9)	0.0146 (4)	0.7643 (2)	0.0609 (16)	
C23	0.7906 (10)	−0.0257 (4)	0.7157 (2)	0.0700 (19)	
H23	0.717649	−0.075398	0.699742	0.084*	
C21	0.5685 (10)	0.1086 (4)	0.6829 (2)	0.0678 (18)	
C17	0.3068 (10)	0.4240 (4)	0.63871 (19)	0.0628 (17)	
H17A	0.239637	0.487146	0.645588	0.075*	
H17B	0.388308	0.410274	0.667678	0.075*	
C13	0.5267 (10)	0.7203 (3)	0.65338 (19)	0.0639 (17)	
H13A	0.656407	0.720735	0.642957	0.077*	
H13B	0.522493	0.722761	0.690684	0.077*	
C24	0.9502 (10)	−0.0553 (4)	0.7415 (2)	0.0657 (18)	
H24	0.980162	−0.124614	0.743378	0.079*	
C15	0.3828 (10)	0.2534 (4)	0.5714 (2)	0.073 (2)	
H15A	0.302064	0.266210	0.542151	0.088*	
H15B	0.452586	0.191077	0.564583	0.088*	
C27	0.8484 (11)	0.1468 (4)	0.7376 (2)	0.074 (2)	
H27	0.815831	0.215889	0.737288	0.089*	
C26	1.0093 (12)	0.1159 (4)	0.7628 (2)	0.085 (2)	
H26	1.081991	0.165116	0.779177	0.102*	
C14	0.5154 (10)	0.3417 (4)	0.5778 (2)	0.076 (2)	
H14A	0.606069	0.325097	0.604224	0.091*	
H14B	0.581967	0.352980	0.545812	0.091*	
C12	0.2334 (11)	0.8264 (4)	0.6508 (2)	0.080 (2)	
H12A	0.178361	0.885510	0.634783	0.120*	
H12B	0.233870	0.835415	0.687461	0.120*	
H12C	0.162330	0.766401	0.642152	0.120*	
C28	1.2434 (10)	−0.0176 (5)	0.7894 (2)	0.083 (2)	
H28A	1.255020	−0.090935	0.787757	0.125*	
H28B	1.243211	0.003861	0.824784	0.125*	
H28C	1.346502	0.013623	0.771844	0.125*	
C18	0.1332 (11)	0.1541 (4)	0.6121 (3)	0.086 (2)	
H18A	0.071686	0.141200	0.644297	0.129*	
H18B	0.198158	0.093334	0.601357	0.129*	
H18C	0.042429	0.172378	0.586578	0.129*	

### 4-{11-Carboxy-7-fluoro-2-methyl-10-oxo-4-oxa-1-azatricyclo[7.3.1.0{5,13}]trideca-5,7,9(13),11-tetraen-6-yl}-1-methylpiperazin-1-ium 4-methylbenzoate Atomic displacement parameters (Å^2^)

**Table:**

	*U*^11^	*U*^22^	*U*^33^	*U*^12^	*U*^13^	*U*^23^
F1	0.124 (4)	0.0551 (17)	0.072 (2)	−0.009 (2)	0.020 (2)	−0.0210 (15)
O4	0.098 (4)	0.0453 (17)	0.053 (2)	0.002 (2)	−0.007 (2)	−0.0014 (15)
O3	0.079 (3)	0.079 (2)	0.048 (2)	−0.001 (3)	0.003 (2)	0.0036 (18)
N1	0.060 (4)	0.044 (2)	0.049 (2)	0.000 (2)	−0.004 (2)	−0.0011 (17)
O5	0.096 (4)	0.050 (2)	0.097 (3)	0.015 (2)	−0.025 (3)	−0.0044 (19)
O2	0.102 (4)	0.077 (2)	0.066 (3)	−0.015 (3)	−0.006 (3)	0.020 (2)
O6	0.119 (5)	0.057 (2)	0.127 (4)	0.012 (2)	−0.065 (4)	−0.019 (2)
N3	0.068 (4)	0.044 (2)	0.066 (3)	0.001 (2)	−0.011 (3)	−0.0008 (19)
N2	0.097 (5)	0.042 (2)	0.074 (3)	0.013 (3)	0.031 (3)	0.005 (2)
O1	0.138 (5)	0.052 (2)	0.083 (3)	0.000 (3)	−0.006 (3)	0.009 (2)
C3	0.039 (4)	0.065 (3)	0.055 (3)	0.001 (3)	−0.001 (3)	0.003 (2)
C2	0.044 (4)	0.049 (3)	0.060 (3)	0.001 (3)	−0.003 (3)	0.004 (2)
C4	0.041 (4)	0.050 (3)	0.054 (3)	0.000 (3)	0.001 (3)	−0.002 (2)
C7	0.058 (4)	0.041 (2)	0.060 (3)	0.004 (3)	0.008 (3)	0.000 (2)
C9	0.050 (4)	0.043 (2)	0.048 (3)	0.002 (3)	−0.002 (3)	−0.002 (2)
C8	0.056 (4)	0.048 (2)	0.047 (3)	0.006 (3)	0.003 (3)	−0.001 (2)
C10	0.052 (4)	0.046 (3)	0.065 (3)	0.003 (3)	−0.008 (3)	0.002 (2)
C11	0.074 (5)	0.048 (3)	0.045 (3)	−0.003 (3)	−0.005 (3)	−0.006 (2)
C6	0.065 (5)	0.049 (3)	0.060 (4)	−0.001 (3)	0.008 (3)	−0.014 (3)
C16	0.064 (5)	0.049 (3)	0.066 (3)	0.010 (3)	−0.001 (3)	0.001 (3)
C22	0.064 (5)	0.047 (3)	0.058 (3)	0.001 (3)	−0.011 (3)	0.000 (2)
C5	0.060 (5)	0.059 (3)	0.053 (3)	0.002 (3)	0.003 (3)	−0.008 (2)
C1	0.066 (5)	0.063 (3)	0.066 (4)	−0.002 (4)	−0.006 (4)	0.015 (3)
C25	0.059 (5)	0.070 (3)	0.054 (3)	0.003 (3)	−0.007 (3)	−0.004 (3)
C23	0.082 (6)	0.048 (3)	0.080 (4)	0.000 (3)	−0.029 (4)	−0.007 (3)
C21	0.079 (6)	0.051 (3)	0.073 (4)	−0.008 (4)	−0.012 (4)	0.000 (3)
C17	0.084 (5)	0.047 (3)	0.057 (3)	0.005 (3)	0.004 (3)	0.001 (3)
C13	0.087 (5)	0.054 (3)	0.050 (3)	−0.001 (3)	−0.012 (3)	0.003 (2)
C24	0.078 (6)	0.055 (3)	0.064 (3)	0.006 (3)	−0.002 (4)	−0.004 (3)
C15	0.093 (6)	0.052 (3)	0.076 (4)	0.009 (3)	0.010 (4)	−0.008 (3)
C27	0.090 (6)	0.051 (3)	0.083 (4)	0.002 (4)	−0.018 (4)	−0.010 (3)
C26	0.091 (6)	0.063 (3)	0.102 (5)	−0.004 (4)	−0.029 (5)	−0.015 (3)
C14	0.091 (6)	0.045 (3)	0.092 (4)	0.010 (3)	0.025 (4)	−0.002 (3)
C12	0.100 (7)	0.081 (4)	0.060 (4)	0.014 (4)	0.011 (4)	−0.004 (3)
C28	0.069 (6)	0.097 (4)	0.083 (4)	0.009 (4)	−0.019 (4)	−0.011 (3)
C18	0.089 (6)	0.063 (3)	0.108 (5)	−0.015 (4)	−0.019 (4)	−0.010 (3)

### 4-{11-Carboxy-7-fluoro-2-methyl-10-oxo-4-oxa-1-azatricyclo[7.3.1.0{5,13}]trideca-5,7,9(13),11-tetraen-6-yl}-1-methylpiperazin-1-ium 4-methylbenzoate Geometric parameters (Å, º)

**Table:**

F1—C6	1.365 (5)	C16—H16B	0.9700
O4—C8	1.362 (5)	C16—C17	1.512 (8)
O4—C13	1.426 (6)	C22—C23	1.392 (7)
O3—C3	1.269 (6)	C22—C21	1.498 (8)
N1—C9	1.388 (6)	C22—C27	1.371 (8)
N1—C10	1.337 (6)	C5—H5	0.9300
N1—C11	1.472 (6)	C25—C24	1.363 (7)
O5—C21	1.264 (6)	C25—C26	1.379 (8)
O2—H2	0.8200	C25—C28	1.498 (8)
O2—C1	1.323 (6)	C23—H23	0.9300
O6—C21	1.231 (6)	C23—C24	1.382 (9)
N3—H3	0.9800	C17—H17A	0.9700
N3—C16	1.488 (6)	C17—H17B	0.9700
N3—C15	1.488 (7)	C13—H13A	0.9700
N3—C18	1.481 (7)	C13—H13B	0.9700
N2—C7	1.389 (6)	C24—H24	0.9300
N2—C17	1.449 (7)	C15—H15A	0.9700
N2—C14	1.458 (6)	C15—H15B	0.9700
O1—C1	1.202 (6)	C15—C14	1.503 (8)
C3—C2	1.415 (6)	C27—H27	0.9300
C3—C4	1.453 (6)	C27—C26	1.387 (9)
C2—C10	1.371 (6)	C26—H26	0.9300
C2—C1	1.487 (7)	C14—H14A	0.9700
C4—C9	1.400 (7)	C14—H14B	0.9700
C4—C5	1.394 (6)	C12—H12A	0.9600
C7—C8	1.396 (6)	C12—H12B	0.9600
C7—C6	1.396 (7)	C12—H12C	0.9600
C9—C8	1.402 (6)	C28—H28A	0.9600
C10—H10	0.9300	C28—H28B	0.9600
C11—H11	0.9800	C28—H28C	0.9600
C11—C13	1.505 (7)	C18—H18A	0.9600
C11—C12	1.509 (9)	C18—H18B	0.9600
C6—C5	1.350 (7)	C18—H18C	0.9600
C16—H16A	0.9700		
			
C8—O4—C13	114.0 (4)	C24—C25—C28	121.2 (5)
C9—N1—C11	119.3 (4)	C26—C25—C28	121.9 (6)
C10—N1—C9	120.4 (4)	C22—C23—H23	119.3
C10—N1—C11	120.1 (4)	C24—C23—C22	121.4 (5)
C1—O2—H2	109.5	C24—C23—H23	119.3
C16—N3—H3	108.0	O5—C21—C22	116.8 (5)
C15—N3—H3	108.0	O6—C21—O5	124.5 (6)
C15—N3—C16	110.6 (4)	O6—C21—C22	118.7 (5)
C18—N3—H3	108.0	N2—C17—C16	109.3 (4)
C18—N3—C16	111.0 (5)	N2—C17—H17A	109.8
C18—N3—C15	111.3 (4)	N2—C17—H17B	109.8
C7—N2—C17	123.7 (4)	C16—C17—H17A	109.8
C7—N2—C14	122.8 (4)	C16—C17—H17B	109.8
C17—N2—C14	113.3 (4)	H17A—C17—H17B	108.3
O3—C3—C2	122.5 (4)	O4—C13—C11	111.5 (4)
O3—C3—C4	121.0 (4)	O4—C13—H13A	109.3
C2—C3—C4	116.5 (4)	O4—C13—H13B	109.3
C3—C2—C1	122.1 (5)	C11—C13—H13A	109.3
C10—C2—C3	120.0 (4)	C11—C13—H13B	109.3
C10—C2—C1	117.9 (4)	H13A—C13—H13B	108.0
C9—C4—C3	120.4 (4)	C25—C24—C23	121.7 (5)
C5—C4—C3	121.2 (5)	C25—C24—H24	119.1
C5—C4—C9	118.4 (4)	C23—C24—H24	119.1
N2—C7—C8	121.1 (4)	N3—C15—H15A	109.5
N2—C7—C6	122.9 (4)	N3—C15—H15B	109.5
C6—C7—C8	116.0 (4)	N3—C15—C14	110.8 (4)
N1—C9—C4	119.3 (4)	H15A—C15—H15B	108.1
N1—C9—C8	119.8 (4)	C14—C15—H15A	109.5
C4—C9—C8	120.8 (4)	C14—C15—H15B	109.5
O4—C8—C7	118.5 (4)	C22—C27—H27	119.6
O4—C8—C9	121.0 (4)	C22—C27—C26	120.8 (5)
C7—C8—C9	120.5 (4)	C26—C27—H27	119.6
N1—C10—C2	123.3 (4)	C25—C26—C27	122.2 (6)
N1—C10—H10	118.3	C25—C26—H26	118.9
C2—C10—H10	118.3	C27—C26—H26	118.9
N1—C11—H11	108.5	N2—C14—C15	110.9 (5)
N1—C11—C13	107.6 (4)	N2—C14—H14A	109.5
N1—C11—C12	110.1 (5)	N2—C14—H14B	109.5
C13—C11—H11	108.5	C15—C14—H14A	109.5
C13—C11—C12	113.4 (5)	C15—C14—H14B	109.5
C12—C11—H11	108.5	H14A—C14—H14B	108.0
F1—C6—C7	116.9 (4)	C11—C12—H12A	109.5
C5—C6—F1	118.3 (4)	C11—C12—H12B	109.5
C5—C6—C7	124.8 (4)	C11—C12—H12C	109.5
N3—C16—H16A	109.3	H12A—C12—H12B	109.5
N3—C16—H16B	109.3	H12A—C12—H12C	109.5
N3—C16—C17	111.4 (5)	H12B—C12—H12C	109.5
H16A—C16—H16B	108.0	C25—C28—H28A	109.5
C17—C16—H16A	109.3	C25—C28—H28B	109.5
C17—C16—H16B	109.3	C25—C28—H28C	109.5
C23—C22—C21	121.0 (5)	H28A—C28—H28B	109.5
C27—C22—C23	117.0 (6)	H28A—C28—H28C	109.5
C27—C22—C21	122.0 (5)	H28B—C28—H28C	109.5
C4—C5—H5	120.3	N3—C18—H18A	109.5
C6—C5—C4	119.3 (5)	N3—C18—H18B	109.5
C6—C5—H5	120.3	N3—C18—H18C	109.5
O2—C1—C2	114.7 (5)	H18A—C18—H18B	109.5
O1—C1—O2	121.0 (5)	H18A—C18—H18C	109.5
O1—C1—C2	124.3 (5)	H18B—C18—H18C	109.5
C24—C25—C26	116.8 (6)		
			
F1—C6—C5—C4	176.0 (5)	C10—N1—C11—C12	84.3 (6)
O3—C3—C2—C10	−179.7 (6)	C10—C2—C1—O2	178.9 (6)
O3—C3—C2—C1	0.0 (10)	C10—C2—C1—O1	−0.8 (10)
O3—C3—C4—C9	−178.3 (6)	C11—N1—C9—C4	177.4 (5)
O3—C3—C4—C5	−0.1 (9)	C11—N1—C9—C8	−6.0 (9)
N1—C9—C8—O4	0.7 (9)	C11—N1—C10—C2	−175.3 (6)
N1—C9—C8—C7	179.7 (6)	C6—C7—C8—O4	−179.4 (5)
N1—C11—C13—O4	−56.6 (6)	C6—C7—C8—C9	1.6 (9)
N3—C16—C17—N2	56.2 (6)	C16—N3—C15—C14	54.8 (6)
N3—C15—C14—N2	−54.6 (6)	C22—C23—C24—C25	−1.9 (10)
N2—C7—C8—O4	0.7 (9)	C22—C27—C26—C25	0.3 (10)
N2—C7—C8—C9	−178.3 (6)	C5—C4—C9—N1	179.4 (5)
N2—C7—C6—F1	2.9 (10)	C5—C4—C9—C8	2.8 (9)
N2—C7—C6—C5	−178.5 (6)	C1—C2—C10—N1	178.6 (6)
C3—C2—C10—N1	−1.7 (10)	C23—C22—C21—O5	164.6 (6)
C3—C2—C1—O2	−0.8 (9)	C23—C22—C21—O6	−17.5 (9)
C3—C2—C1—O1	179.6 (7)	C23—C22—C27—C26	1.2 (9)
C3—C4—C9—N1	−2.4 (9)	C21—C22—C23—C24	177.0 (6)
C3—C4—C9—C8	−179.0 (5)	C21—C22—C27—C26	−176.2 (6)
C3—C4—C5—C6	−177.9 (6)	C17—N2—C7—C8	44.6 (9)
C2—C3—C4—C9	0.9 (9)	C17—N2—C7—C6	−135.3 (6)
C2—C3—C4—C5	179.1 (6)	C17—N2—C14—C15	57.0 (7)
C4—C3—C2—C10	1.1 (9)	C13—O4—C8—C7	155.4 (5)
C4—C3—C2—C1	−179.2 (6)	C13—O4—C8—C9	−25.6 (8)
C4—C9—C8—O4	177.2 (5)	C24—C25—C26—C27	−2.5 (10)
C4—C9—C8—C7	−3.8 (9)	C15—N3—C16—C17	−56.0 (6)
C7—N2—C17—C16	118.4 (6)	C27—C22—C23—C24	−0.4 (9)
C7—N2—C14—C15	−118.4 (6)	C27—C22—C21—O5	−18.1 (8)
C7—C6—C5—C4	−2.6 (10)	C27—C22—C21—O6	159.9 (6)
C9—N1—C10—C2	0.2 (9)	C26—C25—C24—C23	3.3 (9)
C9—N1—C11—C13	32.8 (8)	C14—N2—C7—C8	−140.5 (6)
C9—N1—C11—C12	−91.2 (6)	C14—N2—C7—C6	39.6 (10)
C9—C4—C5—C6	0.3 (9)	C14—N2—C17—C16	−57.0 (7)
C8—O4—C13—C11	54.5 (6)	C12—C11—C13—O4	65.4 (6)
C8—C7—C6—F1	−177.0 (5)	C28—C25—C24—C23	−176.2 (6)
C8—C7—C6—C5	1.6 (10)	C28—C25—C26—C27	177.0 (6)
C10—N1—C9—C4	1.9 (9)	C18—N3—C16—C17	180.0 (4)
C10—N1—C9—C8	178.5 (5)	C18—N3—C15—C14	178.6 (5)
C10—N1—C11—C13	−151.6 (5)		

### 4-{11-Carboxy-7-fluoro-2-methyl-10-oxo-4-oxa-1-azatricyclo[7.3.1.0{5,13}]trideca-5,7,9(13),11-tetraen-6-yl}-1-methylpiperazin-1-ium 4-methylbenzoate Hydrogen-bond geometry (Å, º)

**Table:**

*D*—H···*A*	*D*—H	H···*A*	*D*···*A*	*D*—H···*A*
O2—H2···O3	0.82	1.76	2.522 (6)	155
N3—H3···O5	0.98	1.63	2.610 (6)	175
N3—H3···O6	0.98	2.51	3.125 (6)	121
C11—H11···O6^i^	0.98	2.28	3.147 (6)	147
C12—H12*C*···O3^ii^	0.96	2.52	3.308 (8)	139
C13—H13*A*···O3^iii^	0.97	2.40	3.302 (8)	154
C14—H14*B*···F1	0.97	2.30	2.785 (6)	110
C15—H15*B*···O1^iv^	0.97	2.58	3.268 (7)	128
C16—H16*B*···O2^ii^	0.97	2.55	3.277 (7)	131
C18—H18*C*···F1^v^	0.96	2.54	3.279 (8)	134
C28—H28*B*···O2^vi^	0.96	2.60	3.481 (7)	153

Symmetry codes: (i) *x*, *y*+1, *z*; (ii) *x*−1/2, −*y*+3/2, −*z*+1; (iii) *x*+1/2, −*y*+3/2, −*z*+1; (iv) *x*, *y*−1, *z*; (v) *x*−1/2, −*y*+1/2, −*z*+1; (vi) −*x*+3/2, −*y*+1, *z*+1/2.
